# Ordering Knowledge in the Markers of Psychiatric/Mental Disorders

**DOI:** 10.3390/jcm11020284

**Published:** 2022-01-06

**Authors:** Napoleon Waszkiewicz

**Affiliations:** Department of Psychiatry, Medical University of Białystok, Plac Brodowicza 1, 16-070 Choroszcz, Poland; napoleonwas@yahoo.com

## 1. Introduction

The Special Issue “Advances in Markers of Psychiatric Disorders” (https://www.mdpi.com/journal/jcm/special_issues/Psychiatric_Disorders, accessed on 20 December 2021) presents a novel point of view and an update of the knowledge on the markers of psychiatric/mental disorders. The main thread of the described markers is based on the process of (neuro)inflammation.

## 2. Inflammatory and Seasonal Inflammatory Markers

It seems that the inflammatory characteristic of the onset of various psychiatric illnesses (schizophrenia (SCH), anxiety (ANX), or affective disorders, such as major depression (MDD) and bipolar disorder (BD), as described by the authors of the Special Issue [1,2,3,4,5,6]) is similar, which seems to be due to the hyperactivated hypothalamic–pituitary–adrenal stress (HPA) axis. The differences between psychotic, anxiety, and affective disorders in the inflammatory state appear to start with a decrease in HPA activity. Peripheral cytokine studies suggest that the proinflammatory Th1 (and subsequent Th17) response in depression is dominant, whereas the anti-inflammatory Th2 immunophenotype/response characterizes schizophrenia’s immunity. In anxiety disorders, there is also a serious hyperactivation of the proinflammatory state, which may be due to the increased activity of the HPA axis. In anxiety behaviour, the Th17 response may be crucial, and the observed Th1–Th17 engagement may be due to the HPA hyperactivation and mood changes that accompany anxiety [5,6,7,8].

An earlier study has shown a seasonal mood decrease towards depression [9]. The seasonal mood and anxiety disorders have been suggested to be due to the seasonal air and blood pressure and baroreceptor reflex imbalance, and hyperbaric oxygen therapy sessions have been shown to have similar positive results to those of psychotherapy [10]. Research has also shown a relationship between symptoms of depression and changes not only in air pressure, but also in air humidity, which are more frequent in the autumn–winter and early spring season and, therefore, during the time of the highest prevalence of seasonal depression and catarrhal infection, flu, etc. Symptoms of seasonal catarrhal infection (and/or flu), according to the “sickness behaviour” theory, are similar to depressive symptoms. It is, therefore, possible that the increase in infection caused by a potential pathogen, e.g., a viral one (intracellular pathogen), which spreads more easily in the autumn–winter time due to the higher humidity (droplet infection) [11], may stimulate an inflammatory response of the Th1 type (increased interleukin-6-IL-6, C Reactive Protein (CRP), tumour necrosis factor (TNF)) with a subsequent decrease in the activity of excitatory amino acids, e.g., glutamatergic (GL) and modulation of dopamine (DA), serotonin (5HT), noradrenalin (NA), acetylcholine (ACh), etc., action. The increased cytokine levels may potentially reduce frontal brain activity, which may mimic depressive symptoms in vulnerable patients. High values of IL-6, CRP, and TNF may also be involved in or be the reason for treatment-resistant depression (TRD) and more somatic symptoms, but they may be used to predict the level of depressive response in post-COVID-19 patients [1,3,12]. Higher air humidity (the concentration of water vapour present in the air) around depressive persons were compared to non-depressive persons, and correlations between humidity with symptoms of depression were found, whereas no other weather condition difference, nor its correlation with depressive symptoms, were noted (Białystok Plus study described in [13,14]; meteorological data in preparation). Such a drop in one’s mood may occur not only in the population of those with a higher risk of depression (depressive immunophenotype) but also in people susceptible to schizophrenia [15], which can mimic negative symptoms. A significant increase in hospital admissions due to schizophrenia in the spring and summer is also known [16]. Some interesting influences of temperature, barometric, sunshine, and humidity changes on the symptoms of psychosis have also been described [17]. It seems that, in order to maintain Th1–Th2 immune homeostasis during an infection-related increased Th1 response, the body increases the Th2 response that dominates when the Th1 response declines post-seasonally (post-infection time). Then, the hyperactivation of Th2 is similar to the response that is triggered during a parasitic (extracellular) infection and an allergic reaction with the skin changes predominance, and these were found in schizophrenia [18,19]. In those who are genetically and biologically susceptible to psychosis (psychotic immunophenotype), the Th2 response may be tangibly activated, which can result in excessive glutamatergic stimulation (and the modulation of other neurotransmitters) and subsequent psychosis. Some potential schizophrenia immunomarkers may include transforming growth factor (TGF), interferon (IFN), or other cytokines from the Th2 response or cytokines involved in the Treg tolerance (IL-10), which leave at increased levels when Th1 declines. Interleukine-1, IL-6, and TNF are reduced during antipsychotic treatment, or their decrease can be the result of the reduced activity of the HPA axis as psychosis and its related fear decline [4]. The literature also shows that seasonal temperature changes (increase) may modulate mood changes from a depressive state to a manic state [9], which may potentially/partly be due to the temperature-related changes to humidity and/or barometric changes [17]. The above-mentioned changes in mental health may also be due to the wind and barometric and/or air and water pollution changes [20]. Anxiety and its related HPA axis activation are also linked with atmospheric and seasonal changes. Moreover, the HPA axis activation and its related anxiety are presented in most (up to ~80%) mood and psychotic disorders [20,21,22]. This is in line with recent findings that the anxiety-related Th17 cell response can be induced by the combination of IL-6 and TGF cytokines [7,23] and, therefore, by these cytokines, which seem to dominate in the depressive and psychotic states, respectively, and can be influenced by environmental factors/toxins [23]. It was also reported that the microbiota (especially extracellular intestinal bacteria/fungi) might influence the Th17 response and determine the pathogenesis of rheumatoid diseases [23] that often accompany stress symptoms [24], or it might be that Th17 could mimic such infection. The inflammatory state, which is caused by meteorological changes and the air pollution and infection related to these changes, may subsequently increase the disruption of the lung’s innate immune system through the epithelial barrier, antigen-presenting cells, and protein/cytokine response [11,25]. Humidity in different parts of the world can vary significantly, e.g., depending on the proximity to water, temperature, or altitude [20]. It seems, however, that the absolute level of humidity might not be completely responsible for the entry of pathogens into the body and the subsequent immunological changes, but rather, humidity seasonal changes could be more responsible. Therefore, we can assume that further studies need to find the pathogen (microbial and/or air pollution) that causes seasonal humidity-related psychiatric changes. Such a pathogen load in the body may be a future potential marker of the psychiatric state.

## 3. Stress Axis Markers

It is worthwhile to check the markers of psychiatric disorders when the activation of the stress axis is not at its peak. Therefore, potential trait markers may be found after the partial normalization of the state markers of acute stress/anxiety, e.g., cortisol, immunoglobulin A, alpha-amylase, chromogranin A, Fibroblast Growth Factor 2, and other immunological proteins [5,6,26,27]. The HPA axis mediates the endocrine responses to stress, modulating the secretion of glucocorticoids (e.g., cortisol), which, in turn, modulates the neuroinflammation and its markers [28]. It has also been described that the HPA axis markers of IL-1, IL-6, and TNF can exert their mood-affecting properties through the inhibition of the neurogenesis/synaptogenesis [1], which can predispose people to various psychiatric illnesses.

## 4. Oxidative Stress Markers

Together with the increased pro-inflammatory state, increased oxidative stress markers in depression with its related compensatory increase in the antioxidant enzymes/markers, such as catalase (CAT), superoxide dismutase (SOD) and myeloperoxidase, have also been observed [1]. This is nothing unusual, as oxidative stress is used by inflammatory cells to fight infection, and its values correlate with each other [1,8]. In schizophrenia, the transformation of the Th1 to Th2 response might reduce the antioxidant (CAT, SOD, or peroxidase) response or deplete antioxidant enzymes [4,8,29]. Moreover, a study by Koweszko and colleagues [30] found that there was a positive relationship between the number and intensity of suicide attempts and advanced oxidation protein products (AOPPs), advanced glycation end products (AGE), and a lack of CAT activity in a group of psychiatric inpatients who were hospitalised due to various psychiatric diagnoses. The widely known potential markers of suicide behaviours, such as blood lipids, which may also change due to the oxidative stress reaction, were described by Kułak-Bejda and colleagues [31].

## 5. Other Immune-Related Markers

Some studies of this Special Issue also showed the positive effects of psychotherapy on the peripheral marker of neuroplasticity as a brain-derived neurotrophic factor (BDNF) [32] and on some inflammatory proteins, such as IL-6 and serum amyloid alpha, but not to other cytokines, such as IL-7, IL-8, IL-15, and IL-17 [12]. This suggests that (neuro)inflammation is associated with the somatic symptoms of depression and non-response to psychological therapy.

Some other potential markers of this Special Issue include the epidermal growth factor (EGF) at the onset of the exacerbation of depressive or hypomanic/manic episodes [33] and neutrophil-to-lymphocyte (NLR) ratio, which has been suggested for differentiating bipolar from unipolar depression [34]. In autism spectrum disorder (ASD), a physiological biomarker collected from a wearable sensor’s data was found to be helpful in its differential diagnosis [35], and some augmentations were suggested to help in its treatment [36]. Earlier studies have also suggested that mental symptoms, such as rigidity, stereotypical behaviour that might also be due to the inhibiting properties of the inflammatory state on the brain in persons suffering from autism spectrum disorders, could be connected to inflammation. These persons might have a potential benefit from treatment with the immune-modulating parasite, Trichuris suis ova, or from soaking in a hot tub (>38 °C) [37], which showed that environmental/atmospheric and pathogen changes to the mental state might also occur through their influence on the immunity state and finally on the mood.

Dementia studies showed that the serum YKL-40 protein seemed to be a more useful biomarker in early mild cognitive impairment (MCI) diagnostics, whereas t-tau was a marker of progress of prodromal states in mild Alzheimer’s disease (AD) [38]. However, other studies of serum proteins found that the serum YKL-40 was the only protein (of Aβ42/Aβ40, YKL-40, and t-tau) that was still higher in vascular dementia patients even after 4 weeks of hospital treatment, suggesting the usefulness of YKL-40 for vascular dementia diagnostics [39]. These dementia markers are also immune-related proteins and co-occur with oxidative stress damage, even in saliva [40]. Another study found higher d-serine levels in the serum and cerebrospinal fluid (CSF) of persons suffering from AD [41]. Antczak and colleagues [42] showed that transcranial magnetic stimulation (TMS) had some possibilities for improving the diagnostic accuracy and treatment of dementia, whereas the salivary immunological markers—sTNF-RII and sIgA—might be possible pain markers in dementia [43]. Another study showed that a dual-task test might be a helpful tool in differentiating patients with depression, MCI, and dementia [44].

The state of cognition and its high value for the functioning of patients with cognitive diseases such as dementia, delirium, schizophrenia, etc., is widely known. Krzystanek and colleagues [45] showed that cognitive training facilitated by a smartphone-based application might have the potential to improve cognitive markers/functions in individuals with schizophrenia.

Addiction marker studies presented in this Special Issue show that molecules smaller than 40 kDa, which can easily translocate from blood to saliva, are rather preferred as salivary alcohol markers [46] and changes in the urinary activity of the lysosomal enzyme, hexosaminidase, may be a potential harmful marker regarding drinking alcohol in those who have lost their lives due to ethanol poisoning [47]. These alcohol markers are the result of the inflammatory state’s impact on the metabolism.

## 6. Neuroimaging/Neurotransmitter Markers

Nowadays, in neuroimaging studies, except for a positive [18F] florbetapir-positron emission tomography (PET) signal for detecting increased β-amyloid levels in Alzheimer’s disease, no other specific markers of this mental disorder have been established to date [48]. This Special Issue’s Editorial (see [48]) shows that the atrophy of some brain areas may be compensated by their increased activity. Schizophrenia is generally characterized by the greatest brain atrophy (with ventricular enlargement) and the hyperactivation of the ventromedial prefrontal cortex (vmPFC), the cingulum cortex, and the temporal cortex, whereas the dorsolateral prefrontal cortex (dlPFC) is thinner and shows less activity. In BD, the atrophy of the ventrolateral prefrontal cortex (vlPFC; with its hyperactivation), bigger anterior cingulate cortex (Acc), insula and globus pallidus, as well as a specific hyperintense white matter frontal signal was found. In MDD, a decreased hippocampal, medial, and lateral frontal volume was found, accompanied by an increase in the connection/activity of the prefrontal cortex with the amygdala and a decrease in the activity/connection of the prefrontal cortex with the superior temporal and insular cortexes. Of the ANX disorders, specific changes were described in obsessive compulsive disorder (OCD), where a decreased volume of the orbitofrontal cortex (OFC) was accompanied by its increased activity/metabolic state and a higher thalamus and striatum volume; in panic disorder (PD), there was increased midbrain and brain stem volume; in posttraumatic stress disorder (PTSD), there was decreased amygdala volume; in generalised anxiety disorder (GAD), there was increased activity of vlPFC and amygdala [48].

As different known neurotransmitters can modulate each other, e.g., DA, 5HT, GL, etc., various theories with neuroimaging changes/markers have appeared in the literature [49,50,51]. Of them, glutamate seems to be the most important neurotransmitter, as it is present in over 90% of all brain synapses. Hence, in schizophrenia, the widespread problem of hyperactive glutamatergic psychosis may be associated with long-term neurotoxicity and subsequent atrophy of the cerebral cortex that is not able to inhibit (through fasciculi) the mesolimbic pathway thereafter. In depression, the glutamatergic toxicity of psychosis may be locally limited in the frontal cortex, e.g., by the Th1 general response; hence, the character of psychosis may be limited to an effective, mood-congruent character. In addition, the inflammatory state of the prefrontal lobes may be responsible for their decreased activity, which may result in severe somatic symptoms of depression. In the case of mania, white matter hyperintensity may result in disturbances in the connection of the medial prefrontal and lateral regions. This may result in a consequent reduction in the volume of the vlPFC, which is compensated by an increase in its activity with a possible hyperactivation of the surrounding long-cortical neurocircuit ways (through fasciculi uncinate/longitudinal) and the subsequent stimulation of the other areas of the cerebral cortex, e.g., limbic system, including the hippocampus, amygdala, and a thalamus (mammillothalamic tract). Hence, anticonvulsants can be effective in the treatment of manic symptoms. In the manic state, there may also be a dysfunctional prefrontal medial region (as in depression) and a compensatory way to stimulate the dorsolateral prefrontal region may be disrupted by the above-mentioned hyperintensive changes; there may be an additional disinhibition of the limbic/striatum system. As the glutamatergic system modulates GABA activity (e.g., NMDA receptors on GABA neurons; [52]), the deficiency of the glutamatergic inhibitory activity may result in the insufficient release of GABA from the neurons of the cortex (e.g., cingulate cortex) into the striatum and tegmentum and then to an excessive dopamine release.

Inhibitory neurons also have cholinergic activity, which is why cholinolytics can induce delirium (psychosis with excess activity). Delirium symptoms increase at night with the deprivation of visual, hearing, etc., stimuli of the prefrontal cortex and with the increased inflammatory state (increased IL-6, CRP) [52], which can also result in prefrontal activity reduction and the subsequent worsened inhibition of the subcortical psychotic areas. When psychotic symptoms are recurrent, it is also possible that hallucinations may be the result of a flashback-like or persisting perception effect after the potential elimination of a pathogen/substance [53]. However, as in the flashbacks, the concentration of pathogen/substance can re-occur upon the release of its cumulative dose from body organs, and re-hallucinations can be potentiated by the stimulus deprivation in individuals prone to psychosis. Future research needs to clarify whether the accumulation of potential pathogens in tissues and the flashback mechanisms are also possible in mood and psychotic disorders.

## 7. Conclusions

This Special Issue entitled “Advances in Markers of Psychiatric Disorders” provides a lot of new knowledge in this field and will hopefully lead to more discoveries. Overall, these 29 published contributions may strengthen the essential function of markers in psychiatric diagnosis in the literature. I also hope that this area of research will benefit patients and their families. Factors predisposing individuals to the development of psychiatric disorders and their markers are infectious/immune, metabolic, and environmental (e.g., stress, atmospheric, dietetical, etc.) and also (epi)genetic and neoplastic aetiology [54,55]. An example of difficulties in aetiological interpretation in psychiatric research is explaining the formation of mood disorders by only one factor, e.g., dietary changes. If only poor nutrition and dietetical factors were responsible for mood disorders, e.g., depression, then they should potentially not occur after the spring/summer season, when nutritional deficiencies are usually replenished. Various populations and races may have predispositions to react differentially to various factors and in levels of markers, e.g., a rapid normalization/catabolism of elevated markers in children and adolescents [56]. Hence, there is also an urgent need to find markers of psychiatric disorders in these groups. It is also interesting if such an immune Th1–Th2–Th17 interplay during brain development may predispose people to mental disorders and correspond to the neurodevelopmental and/or neurodegeneration theories. A very important point of future research is also to find the pathogen (microbial-viral, air pollution) that influences seasonal humidity-related mental state changes and induces marker production in the body. Similar to antibody-mediated (autoimmune) encephalitis, which until recently was a mysterious and unexplained disease characterised by catatonic symptoms (disturbances in consciousness with psychosis and agitation) [57], much more common mental disorders also/especially need a detailed medical explanation of their symptoms.

Although the progress in markers of mental illness cannot come quickly enough, the future is starting to look brighter, and more markers will soon hopefully be within our grasp.

## Data Availability

Not applicable.
